# Audience reconstructed: social media interaction by BTS fans during live stream concerts

**DOI:** 10.3389/fpsyg.2024.1214930

**Published:** 2024-04-18

**Authors:** Finn Upham, Jin Ha Lee, So Yeon Park

**Affiliations:** ^1^Department of Musicology, RITMO Centre for Interdisciplinary Studies in Rhythm, Time and Motion, University of Oslo, Oslo, Norway; ^2^Information School, University of Washington, Seattle, WA, United States; ^3^Center for Design Research, Stanford University, Stanford, CA, United States

**Keywords:** audience interaction, Twitter, social media, BTS, Kpop, live stream concerts

## Abstract

COVID-19-motivated social distancing made online concerts common practice in 2020 and 2021, with millions logging into streaming sites to see their favorite artists perform in realtime. For some fans, watching alone at home may have been enough, but concert-concurrent surges of social media activity suggest many virtual performance attendees are doing more. To understand why fans would turn their attention from these precious performance streams to social media, we explored Twitter engagement during four live streamed concerts performed by the Kpop group BTS in 2021. In public Tweets sampled by either concert hashtag or a predefined stream of users and keywords, we evaluated patterns in posting rates in relation to concert program events and investigated the content patterns in 1,200 Tweets sampled from four ranges of popularity (number of Retweets during the concert). Across concerts, short “Shout” Tweets surged at the start of songs, while the rate of retweets often fell during musical performances and shot up when BTS was off stage. Content analysis on the subsample found the materials most widely shared were informational or featured concert visuals, mimicking how fans use their phones at in-person concerts. Most original posts received few Retweets and were more personal and expressive of admiration for the performers. Comparison between the samples (concert hashtag vs. stream) also suggests users were strategic in using or omitting official concert hashtags with the strongest differences in the most widely disseminated content. Postings on Twitter during these performances seemed principally directed to fellow fans and audience members, by individuals choosing to share their own excitement and check in with others. By leveraging their existing social media networks, these concert attendees constructed a collective and interactive concert space, connecting with friends and strangers in the crowd and helping each other capture a richer experience than any broadcasting platform currently supports.

## 1 Introduction

The COVID-19 pandemic produced a period in which online broadcasts were the only option for most fans and musicians to meet in concert. In this time, the Kpop phenomenon BTS performed several live streamed shows with hundreds of thousands of simultaneous paying viewers tuning in from their homes around the world (Kiswe, 2021). While fans were excited to see these realtime performances, their attention was not solely focused on the broadcast stream: concurrent activity on social media suggests that many switched between watching the performance and engaging with other fans on alternative platforms. We use Twitter data recorded during four concerts to look at when and how fans used this platform during the broadcast shows and what kind of materials they were sharing. The patterns suggest fans use social networks to recreate many of the activities they would perform as audience members in a live concert, from cheering loudly for favorite songs, to capturing clips for future memories, to basking in the emotional journey together.

## 2 Background

Music concerts are social events, gathering people to watch a performance and share a special experience. The presence of like-minded people adds to the attractiveness of live concerts (Brown and Knox, 2017), bringing a “sense of unity” or *ittaikan* with other audience members as well as between the audience and performer (Tarumi et al., 2017). This social component of concert experiences is often part of people's favorite musical experiences, when they feel connected to friends and strangers (Krause et al., 2020).

How audience members' experiences are shared depends on what the music and venue allow, from occasional rounds of polite applause to continuous writhing and screaming in the mosh pit. These norms of audience behavior during performances still allow individual attendees to be more or less active, according to their own priorities and position in a performance space (Fonarow, 1996; Benzecry and Collins, 2014). This behavior can include conversation at the interval or in the back of the bar, coordinated swaying at the edge of the stage, or enraptured fans watching in determined stillness. An active audience can also be perceived as detrimental to a concertgoer's experience because they can distract from the staged performance (Pitts, 2014; Mulder and Hitters, 2023). Some of the risks of going to live performances come from other attendees and their choices to participate, whether too much or too little. A common point of contention is how audience members use mobile phones at in-person shows. Even audience members who use their mobiles to capture special moments express ambivalence, concerned about how it may impact others in the audience as well as their own engagement in the show (Kjus and Danielsen, 2014).

When music performances are broadcast, audience members' opportunities to see and be seen, hear and be heard, are drastically changed (Charron, 2017). Their contributions to performances depend on what the available technologies allow, from the vague allusions on TV broadcasts like “the nation is watching” (Holt, 2010), to digital avatars jumping around the virtual performance space (Onderdijk et al., 2023). How effectively these mediations allow audience members to feel socially connected is an ongoing topic of research.

### 2.1 Live stream concerts and livechat

Live stream concerts became more common with the social distancing restrictions imposed to control the spread of COVID-19. Lockdown drove musicians and audiences to find new ways of connecting without risking public health (Hansen et al., 2021). Already a common practice for some portions of the internet, these technologies suddenly became relevant to a larger population, including new genres of concertgoers (Rendell, 2021). Most of these streams include an optional text chat function: besides showing the video feed of the performance, a text chat window is available to attendees to share comments in realtime with everyone watching. How such chat functions are used depends on the genre of stream, distribution platform (and what functions it supports, such as reaction emoji and @ing users), the number of people watching the stream, and of course, individuals' inclination to attend to and use these mechanisms of participation.

In a survey of live streamed concert experiences in the first months of the pandemic restrictions, Swarbrick et al. (2019) explored the experience of remote-concert attendees, including impressions of connectedness and social presence with performers and with the audience. Uses of interaction features like making comments in the livefeed chat correlated with greater *kama muta* and social connectedness. From a secondary analysis on the published survey data (Swarbrick, 2021), it seems survey participants varied in their use these features, with only 1 in 5 reporting sharing multiple or detailed comments on their experience for other audience members to see. Still, these few chatters also reported feeling greater connection and shared feeling with the audience, that others were aware of their presence, and that the streaming audience members were active and engaged (see Supplementary material).

An experimental attempt at investigating social connection through this medium assigned participants to attend concerts in a live streamed music festival in the summer of 2020 (Onderdijk et al., 2021). The condition that allowed audience participants to see each other (Zoom room with participants' cameras on) encouraged the impression of social presence; however, participation in the textchat function was too sparse to consider statistically as a mechanism for social connection.

Comparing behavior in FacebookLive chats for classical music and the Dutch popular music genre *levenslied*, both fairly new to live streaming in the early pandemic period (20 March until 17 April 2020), Vandenberg and Berghman (2023) investigated comments on performances that accrued dozens to hundreds per show. These commonly referred to face-to-face interaction practices (flowers, clapping, other audience actions typical during these shows) with few instances of explicit pairwise interaction between participants.

One likely reason for the limited concentration of audience action and interaction observed in these pandemic-oriented studies is technological unfamiliarity. Watching live streamed classical music concerts was a novelty before the COVID-19 lockdown, requiring extraordinary campaigns to engage audience members in a relatively convenient and low-cost activity (Nguyen, 2018). However, there are other communities with established cultures of interaction between performers and viewers over live stream. Twitch.TV is a massively successful streaming platform that has grown many distinct common practices of interaction through the limited medium of textchat (Jodén and Strandell, 2022). When thousands of users are posting to the same live chat, the flood of text messages becomes unintelligible. This cacophony can be reigned in with some coordination strategies, such as *crowdspeak* where viewers opt to share a common short message, making a cascade of posts that make the content visible to viewers and streamer alike (Ford et al., 2017). These messages can be inside jokes, important questions, or descriptors of more typical audience actions, but crowdspeak depends on viewers catching the impulse to emphasize a message in chat and many more seeing this opportunity and joining in. This culture of chat strategies grew in the context of immediate interaction between viewers and the streamer during live videogame playing. Music concerts rarely allow for this kind of continuous interaction, restricting the performers' responses to between pieces or sets, or in many cases, completely ignoring the chat altogether (Rendell, 2021).

From a series of interviews of individual users' experiences of music concert live streams on Twitch.TV, Vandenberg (2022) found many to follow these performances with less attention than face-to-face performances. According to observed behavior and self-report, the participants' interactions with other online users over chat were less intense or affecting than engagement with people in their preexisting social sphere. While the rare direct interaction between viewers seemed to be strongly felt, the medium of live chat made such exchanges slow and awkward and limited to more generic topics than might be discussed between established friends.

The effectiveness of livechats on concert live streams to satisfy audience members' desire to participate and connect seems highly variable. The experience offered by a small audience without a practice of commenting may allow the few active users to feel seen, while the waves of crowdspeak give users a chance to act together. Both extremes are complicated by the fact that messages posted are broadcast to all virtual strangers in the virtual room, including the performers, if they choose to look.

### 2.2 Twitter

Before being renamed to *X* in 2023, Twitter was a free social media platform that allowed registered users to post short (≤280 characters) status updates (*Tweets*) that could also include still images (up to four), gifs, or short video clips. Users also followed other accounts, reading these users' Tweets on their Timeline, and had multiple options for interacting with these posts: by favoriting (*Liking*) Tweets to show approval, sharing an existing Tweet on their own profile and the Timelines of their followers (Retweeting), initiating a dialogue by responding to a Tweet with one of their own (replying), or sharing a Tweet with additional media and commentary (*Quote Tweeting*). Replies and Quote Tweets differed primarily in their visibility on other users' timelines. Replies were not directly shared with the replier's followers unless they also followed the account being replied to, however they allowed for conversation chains to be constructed easily in certain interface views. In contrast, Quote Tweets were immediately visible to all of the quoter's followers and were more awkward for extended exchanges. Twitter's web browser and mobile app interfaces also allowed searches of the Tweet database by text, which encouraged the use of hashtags: distinct short connected text strings starting with a hash symbol (#) that people could add to Tweets about a specific event, topic, or theme. Hashtags allowed non-followers to easily find posts of interest and engage with them on (or off) the platform.

Through the 2010s, *live tweeting* became a popular means of documenting and publicizing political and cultural events (Kjeldsen, 2016). One or several Twitter users would post descriptions or commentary of an ongoing event in realtime, often with an identifiable hashtag to facilitate retrieval by strangers. This microblogging practice has been popular amongst academics, journalists, cultural critics, and activists, making inaccessible events visible to a wider public and sharing information with their particular point of view (Pemmaraju et al., 2017; Reyes-Menendez et al., 2018). Live tweeting to broadcast events also allowed everyday spectators to share their take (Hawthorne et al., 2013; Ji and Raney, 2015), and a study of sports spectators reported their enjoyment of both mediated (live broadcast) and in-person games to increasing with the intensity of engagement on the platform, namely by posting Original Tweets or a combination of Original Tweets and Retweets (Smith et al., 2019).

The observation of live tweeted events on Twitter is more difficult to describe. At the time of data collection for this study (2021), the Twitter platform defaulted to filling users' timelines with mostly chronologically-ordered content from the accounts they followed with some algorithmic alterations (Newton, 2016). Besides adding posts by advertisers, Tweet order could be shifted to prioritize recently popular content within their network of followers, and additional out-of-network popular Tweets may have been offered according to user behavior (Johnson, 2021). Many of these adjustments to chronological presentations of Tweets seem geared toward making interesting current events more visible to users, likely facilitating the propagation of live-tweeting activity. Outside of their Timeline and brute searches on the platform, Twitter also presented users with *Trends* in their noted areas of interests: curated lists of currently hot topics, keywords, and hashtags assessed globally, regionally, and by themes like music or sports.

Twitter began allowing researchers to explore the public Tweet database in 2006 through a licensed API access (Tornes, 2021). Besides research into the network properties of topics (Himelboim et al., 2017) and communities using dedicated hashtags (Chandra et al., 2021), the expression of activity on the platform by remote spectators has been of some interest (Highfield et al., 2013), especially for sports (Hsieh et al., 2012; Lanagan and Smeaton, 2021). Twitter analysis might not have been the fastest way to hear about who scored what goal, however evidence of such dramatic moments in matches suggests that this platform has been used by fans to share their elation in parallel to offline shouts and cheers.

### 2.3 Phones and social media at concerts

Whether the audience is attending concerts face-to-face, over live stream, or through a virtual environment, attendees also have the option to capture and share their experiences outside of these environments. Preparatory, concurrent, and post-concert activity on social media platforms have made these common secondary spaces for concertgoers to extend the event. Pictures and videos of shows are routinely shared in online space, privately and publicly, and in the many shades between.

Tweeting from live concerts has at times been quite controversial. Classical concert spaces have been particularly resistant, and mobile phones and their cameras are still forbidden in some venues (Glitsos, 2018). Tweet Seat concerts, operatic or symphonic performances where a few select audience members were invited to live tweet along, were a seen as daring form of outreach in 2016 (Nguyen, 2018).

Live tweeting and mobile phone recording became common at popular music performances much earlier, bringing events in the concert hall to a large audience through commentary, photos, and video clips. By 2014, audience members had strong opinions about how these technologies changed their experiences both in and out of the venue. Bennett's (2014) interviews of Tori Amos fans found mixed experiences. In addition to the benefits of sharing information with people who could not be there, which strengthens an interested online community, the people sharing also reported some interference with their own engagement with the show. As one participant said: “I definitely don't feel as connected to the moment when I'm texting/tweeting. I try to pause and take in the music and the performance before sending an update.” Using these technologies to communicate with fans outside of the concert space has costs.

More commonly, audience members are taking video and photos for their own private enjoyment as evidence of “being there” and to capture “novel performance moments,” whether or not they are shown to others (Kjus and Danielsen, 2014; Kim and Kwon, 2019). Holding up a phone and pressing a button is less cognitively demanding than formulating and typing up messages to post, and it still captures precious material. The consequence of glowing screens across an audience has been lamented by many (Glitsos, 2018). Still, for some venues, shows, and particularly for shorter audience members, the phone raised high allows for a better view of the action than they could get from standing (personal experience). Whether or not users are recording or reporting on social media, the mobile phone screen is a ubiquitous component of live face-to-face music shows wherever they are allowed.

### 2.4 The active Kpop audience

In the Kpop culture, fans are used to being active audience members. Fans normally shout and sing along, follow bits of choreography, and perform the relevant *fanchant* for many, if not most, songs performed. A fanchant is “a chant that fans recite in unison during the artists' performances consisting of parts of the lyrics, names of the group/members, or other words” (Bhattacharya et al., 2023), and this concert practice requires that fans obtain the official fanchant instructions for the expected set list of the show and practice their part before attending the performance. Derived from the Korean folk music tradition of *Chuimsae* (Takayanagi, 2021), fanchants are an example of the degree of preparation and participation Kpop culture expects of the audience.

Around face-to-face concert events is also a culture of community forming between attendees. Hours-long lines for entering stadium venues and accessing merchandize give these fans time to chat and bond with like-minded people while performing effortful devotion (Benzecry and Collins, 2014). It is also common practice for fans to bring materials like photo cards and trinkets to share with the people they meet at the venue (Guillen, 2022). And like other pop genres, it is normal to watch in-person shows with phones up (when allowed in the venue), capturing photos and videos so that these precious moments can be replayed for personal enjoyment and shared with others (Kjus and Danielsen, 2014; Kim and Kwon, 2019).

The participatory nature of Kpop fandom is also very prominent outside of the concert context. In the fan community for the group BTS, officially called *ARMY*, there are different subgroups or types of fans who dedicate their time to engage with BTS-related content through various activities. These subgroups include “Theory ARMYs” who enthusiastically collect, analyze, and interpret the hidden meanings or symbolism based on BTS' song lyrics, music videos, and other content; “ARTMYs” who express their love of BTS through their artistic creations such as drawing, knitting, crafting, etc.; ARMYs who do dance covers of the BTS songs; and so on (Lee, 2019). These activities are often tied to performed music (Lee, 2018) where fans share what they think of as the meaning of the video content shown during the concert, drawing different scenes from the concert, or creating memes with the different screenshots of the concert footage. This kind of content is actively shared on social media, and some of the accounts gain a substantial following as other fans enjoy and appreciate their content. Through these participatory activities and sharing practices, fans connect with each other and build a sense of community.

BTS's ARMY is an audience used to being recognized and acknowledged by performers during the show. Besides addressing fans directly during performances, Kpop group members talk extensively about their personal investment and affection for fans in non-concert live streams and behind-the-scenes footage. Chat messages from fans during these live streams can turn into an inside joke between the artist and the fans (Ringland et al., 2022). For example, once BTS members pointed out the many live stream chat comments saying “Yoongi Marry Me,” this phrase turned into a meme, and fans began to bring signs with the same message to face-to-face concerts as a playful joke. Kpop groups also create “fan songs” about their fans and the fan-artist relationship with lyrics containing messages directly for their fans. For instance, BTS created the song “2! 3!” as a way to comfort fans during the hardships they were experiencing due to false accusations and attacks from other fan communities at that time. Each year since their debut (excluding their current break), BTS has celebrated the formation of ARMY with *Festa*, a two-week interval of special promotional activities culminating in a distinctly fan-oriented concert (the *Muster*).

From specific concert activities, established social practices, and explicit acknowledgment from their shared focus, ARMY is a music fan community that is very interested in each others' experiences and empowered to work for their collective benefit both in-person and online.

### 2.5 COVID compromises for Kpop shows

When the COVID-19 pandemic put a stop to face-to-face performances, Kpop was more prepared to compensate than most sectors of the global music industry. The leading companies had previously been exploring different ways to use augmented reality (AR), virtual reality (VR), and other technologies to provide high quality “on-tact” (online-contact) events (Kim, 2021). Kpop audiences also had years of experience engaging with live streamed content from these performers on platforms like V Live (Kim et al., 2021). Thus, BTS was able to host online concert events quite successfully in 2020 and 2021.

Several strategies were employed to bring aspects of an audience to the performers for these concerts. In sets built to the scale of stadium stages, sections of the performance spaces were decorated with hundreds of *ARMY bombs*, the light sticks that fans bring to concerts to be part of the Bluetooth-coordinated light show, supposedly representing the absent audience and recreating the typical atmosphere of the concert. Imitations of the sonic presence of an audience were also added through synthesized cheering, and even mixes of fans-submit fanchant recordings were added to the backing tracks for BTS's singing and dancing. At the BTS Map of the Soul ON:E and Sowoozoo muster performances, a prepared group of fans were set up to stream from home into the performance spaces, making a wall or field of faces visibly cheering in near realtime for the group's performances (Lee and Kao, 2023). During these concerts, BTS members mentioned the challenges of performing without a live audience and discussed how the sight of streamed-in ARMYs cheering with their homemade signs made them feel more connected and helped them to stay positive in these difficult conditions.

VenewLive, the ticketed online broadcasting platform supporting these BTS concerts (Kiswe, 2021), offered a built-in live chat function allowing audience members to message everyone following the stream. This chat was open by default but could be hidden with a toggle. As has been observed on Twitch.TV, livechats on streams with massive audiences (≥10,000) quickly become a torrent of messages that cannot support normal chat interactions (Ford et al., 2017). Anecdotal accounts from fans suggested many instead used social media and private communication channels to connect with others watching in realtime. Discord, Zoom, and DM (direct message) groups on many platforms allow for more personally pertinent and manageable sharing of the concert experience while places like Twitter and Tumblr fell in between. These sharing-oriented social media platforms gave fans access to the experiences of known network friends and strangers through reblogging or Retweeting mechanisms and hashtag or topic feeds.

Our choice of looking at online concert activity on Twitter was opportune. This was a platform with highly concentrated online engagement by the followers of BTS (ARMY), and a social media space with a working API which allowed licensed researchers to study online activity. The resultant data is a rare side view into a massive paying remote audience's realtime interactions during a stadium-tour quality concert production.

## 3 Materials and methods: Tweet timing

Twitter activity during four live streamed BTS concerts was collected in realtime to compare with the broadcast concert timelines. Analysis was performed on the timing of Tweets and their content using automated feature description and manual coding of subsamples.

### 3.1 Concerts and concert events

Twitter activity was tracked around four concerts in 2021. The first two concerts were BTS's 2021 Muster, named Sowoozoo, which took place on June 13 and 14. Musters are special celebratory performances with a particular focus on their fan community. The program often features more casual conversations between band members and special productions for songs that would not be taken on tour. The last two concerts were online broadcasts of BTS's Permission to Dance (PTD) Tour: PTD on Stage, performed for broadcast in Seoul on October 24, and PTD in Los Angeles on December 3, performed both for broadcast and to a face-to-face audience, as was newly permitted in the United States with changes to COVID-19 pandemic lockdown restrictions. The concert pairs showed mostly the same program, and presumably attendance to the first broadcast of each was higher than the second.

These concert streams combine different types of content that we collapsed into four categories: Live music, Live talking, Video clips, and Off stage. Besides the sets of songs performed live (Live music), there were intervals of live talking as band members addressed the audience and chatted amongst themselves (Live talking). Some of these intervals of talking were quite long, with performers' comments to the audience before the last set of the songs sometimes running up to 20 min. In these talking intervals, members spoke mostly or entirely in Korean, and these broadcasts offered realtime translations in a few common languages. There were also prepared video sequences dispersed throughout the concert consisting of short clips that allowed for costume and set changes (Video clips). These videos, referred to as “VCRs” in Kpop, often follow the band members through a wordless fantastical narrative. Lastly, there was always a cheering section toward the end of the show when the performers were offstage, and the broadcast feed showed live, recorded, or streamed-in fans while they cheered before the closing set (Off stage).

In addition to the intervals defined from the concert live stream program, 5 min intervals before and after the stream were added to the Off stage category. Live music intervals were marked from the start of each song performance, Live talking from when members began talking after a set of songs ended or with a new programmed conversation activity, Video clips and Off stage sections from the change in broadcast feed. Each show lasted between 2 and 3 h, with 25–37 program events, and 19–23 transitions between these categories of stream content. The relative timing of these events was marked manually from the original concert broadcasts and rebroadcasts.

Online broadcasts always have some delay from the stage side, and viewers receive the broadcast with variable additional delays dependent on their geographical location, internet connecting stability, and the broadcasting platform's resources. It is not possible to align the timing of these program intervals perfectly to the times they were seen by all online viewers. Instead, the concert program timelines were brought into approximate alignment by scanning for mentions of performed music tracks. The event onsets were shifted en mass so that the music intervals started within a few seconds of the first cluster of posts about a song. As such, the alignment of concert intervals with the Tweets compensates for both some portion of viewing time offsets and a representative reaction-to-expression delay on this platform.

### 3.2 Twitter samples

Tweets posted during four live streamed concerts were captured using the Twitter Streaming API, then collected. They were collected and stored by the Center for an Informed Public at the University of Washington. The Streaming API allowed for Tweets to be collected according to predefined monitoring criteria made up of user IDs, keywords, and hashtags (Twitter Developer Platform, 2021). For each Tweet captured, the API logged the text of the post, when it was posted, along with information on Tweets related by Retweet, Quote Tweet, and Reply, and public User Account details for the posting user and users of related Tweets such as their user id, number of followers, and account language. All public Tweets meeting the pre-defined criteria were captured except in cases where rate exceeded limits. Tweets by private accounts and actions like direct messages are not available through the API, nor are actions such as Liking Tweets or views. Additionally, public accounts that explicitly requested exclusion from data collection through common statements in their user descriptions were filtered out prior to analysis (See Supplementary material for details).

For the two Sowoozoo performances, concert Tweets were collected in realtime using exclusively the concert hashtag. After capturing all public Tweets that included the hashtag #SOWOOZOO (ignoring case) in the days around the 2021 Muster, this collection was cut down to an interval of 3.5 h around each concert, 225,934 and 114,724 status updates, respectively. While not all concert-related Tweets used this concert hashtag, this method of sampling captures nearly complete Retweet trajectories for content intentionally associated with the performances, giving a detailed view of network activity within an interested segment of Twitter users.

For the Permission to Dance concerts, the concert hashtags were not used to a comparable degree. The most popular hashtag for the first PTD concert, #PTD_ON_STAGE, occurred 46,321 times, less than half as many as the second Sowoozoo show. During the LA performance, #PTD_ON_STAGE_LA was found in only 7,917 Tweets during the recording interval. This was perhaps due to rumors circulating online about accounts getting suspended for sharing concert feed footage, making attendees shy to tag their content on Twitter. Instead of collecting Tweets with only the concert hashtag, we sampled Twitter activity from a previously-established stream. In 2020, a project at the Center for an Informed Public had defined a select population of BTS-oriented Twitter users (≤5,000) to monitor continuously for activity within this region of Twitter. This population was constructed from a partially random selection of users who followed a few key accounts within the ARMY network, had posting histories focused on Kpop, and matched a minimum rate of posting activity. This group of accounts formed the core of the Stream samples, along with the inconsistently applied concert hashtags and some keywords from other projects. Despite the particular structure of this stream, this cross-section of Twitter still showed a substantial jump in activity during the broadcasts. There were 277,794 and 143,772 status updates collected via the stream through the four-hour intervals covering each PTD broadcast concert, many times the amount captured with the same criteria a week prior (see Supplementary material for more on the off-concert Stream samples). Inspection of subsamples from the concert intervals also found the vast majority to be related to the performances, sufficiently concentrated for quantitative and qualitative analysis. Still, it should be noted that the Stream samples are structured very differently from the Hashtag samples, both by including content that is not posted with an official hashtag, i.e., not intended for a global or commercial audience, and by mostly capturing instances of Tweets passing through a monitored population of longtime Twitter users.

Our interest was primarily in audience actions and interactions in a social network with very high centrality around official accounts. Fan-to-fan engagements can be completely overshadowed by the scale of concurrent responses to accounts with millions of notified followers. To attenuate this type of noise, the samples were filtered for Replies and Retweets of some official accounts, specifically: “@BTS_twt,” “@bts_bighit,” “@BIGHIT_MUSIC,” “@weverseofficial,” “@weverseshop,” “@HYBE_MERCH,” and “@BT21_.” These filtering criteria have differing impacts on the samplings: <4% of posts and unique users were cut from the Hashtag samples, while 15–17% of Tweets and 22–25% of users were removed from the Stream samples. Table 1 reports the final number of Tweets studied per concert, including by type.

**Table 1:** Statistics of the four concert Tweet samples.

**Concert (sample source)**	**Filtered**	**Originals**	**QT&Replies**	**Retweets**	**RTed**	**Users**
SWZ day 1 (Hashtag)	224,733	26,939	8,459	190,998	6,006	111,147
SWZ day 2 (Hashtag)	111,185	18,816	4,093	89,151	5,451	54,457
PTD on stage (Stream)	228,793	5,548	10,555	214,426	9,323	85,374
PTD in LA 4 (Stream)	116,342	4,013	9,636	107,671	8 548	44,130

Posts counted after filtering, number of posts by type, number of unique posts being retweeted (RTed), and total number of unique user accounts (posting and retweeted).

There are four types of status updates captured in these datasets: Original Tweets, Retweets, Quote Tweets, and Replies. Retweets are by far the most common form of posting activity for these samples. Besides scrolling past or just Liking a Tweet, Retweeting is the least effortful interaction a user can make with content they see on their timeline. The action of Retweeting both propagates the Original Post to followers and preserves the material in the user's own posting history. Quote Tweets and Replies are also interactions that can make posts retrievable; however, they are much more complicated to document. Their prominence in the Stream sampled data is concentrated around interactions prompted by large fan accounts in ways that are ambiguous in their relationship to concert content, while the same pattern of platform activity was negligible in the Hashtag sampled datasets. Table 1 collapses these types together, and they are not included in the analysis to follow.

### 3.3 Tweets over time

**Figure 1:**
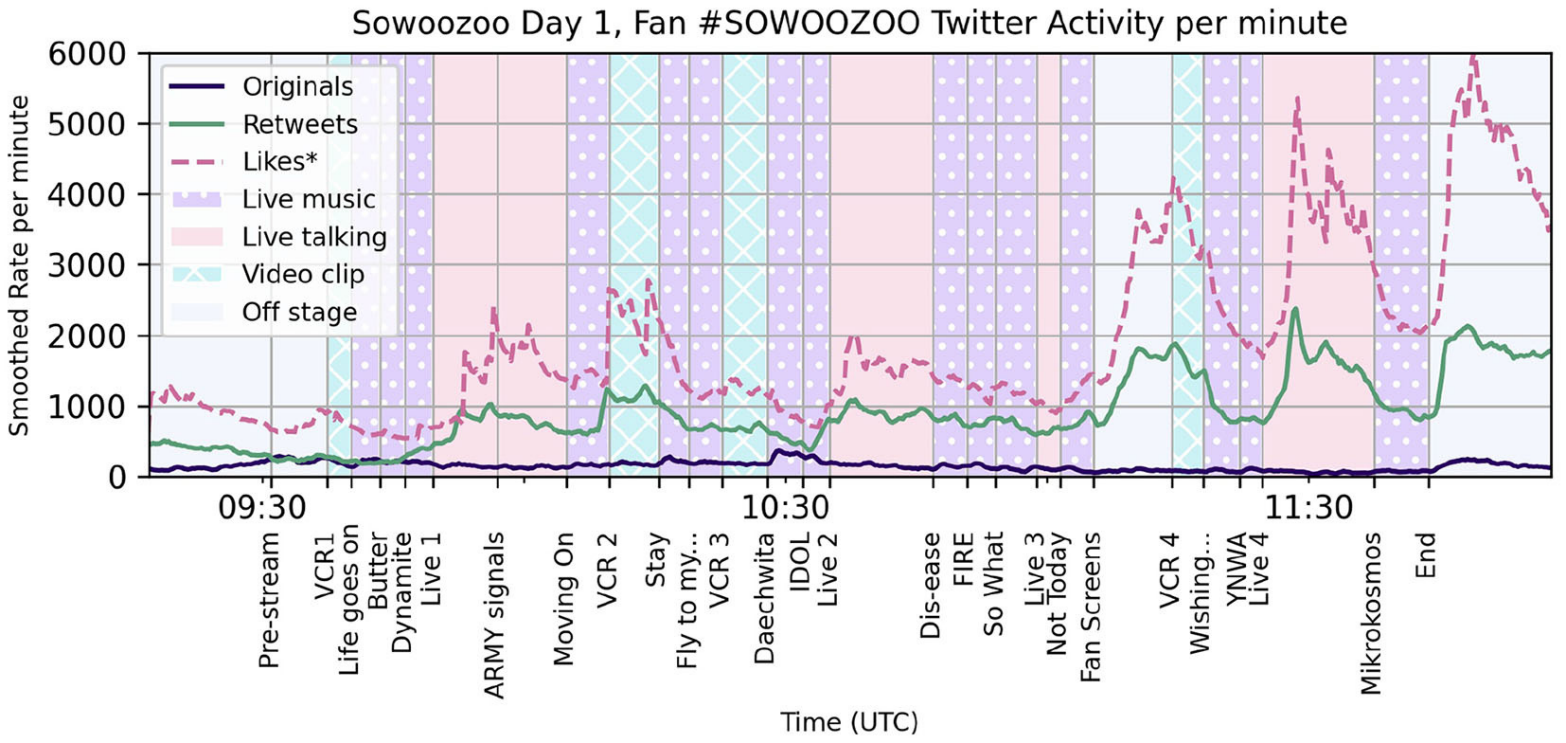
Smoothed minute rate for fan Twitter interactions with #Sowoozoo over the course of the Sowoozoo concert live stream on June 13th, 2021. Retweets and Original Tweet rates counted directly from Hashtag sample Tweets excluding official accounts. Likes rate is estimated from the accumulation on the 1,000 most retweeted Tweets. Concert stream content categories color-coded in background with events labeled at onset on the x-axis, including song titles.

Analysis of the timing of Tweets by type and association was conducted in Python using common libraries and specialized scripts (https://github.com/finn42/Concert_Twt_Open/; https://zenodo.org/doi/10.5281/zenodo.10159761). As described in Section 3.1, the concert program timing was aligned to notable markers in the Tweet dataset, namely the onset of specific tracks. Tweet rates were initially counted in 15 s intervals for dense samples and 60 s intervals for smaller sets such as Retweets of a single post. The smoothed rates per minute seen in Figure 1 are the result of a centered four-point rolling sum, retaining the 15 s hopsize.

The rate of Likes during the first Sowoozoo concert was estimated from the accumulation of Likes on the 1,000 most retweeted Tweets. Total Likes on these posts were counted cumulatively with each recorded Retweet, differenced on the 15 s sample intervals and smoothed like the posting rates. These values are not a complete picture of the Liking actions performed by concert attendees on Twitter during this interval. They are entirely missing Likes on Tweets with the hashtag that were not subsequently publically retweeted within the sampling interval as well as Likes on Tweets that were retweeted <14 times. However, the series' proportions to the other rates reflects the changing number of users attending to their Twitter timelines during the show.

Absolute rates of Tweets cannot be fully modeled from the information available. To consider the impact of concert content on Twitter activity, we instead focus on local shifts in rates between successive segments of the concert program. We defined the *Rate Shift* as the ratio of the average posts per minute through one segment of the concert program, say a set of songs, relative to the average posts per minute through the previous segment. The *Cusp Shift* ratio is a similar comparison made between just the 60 s before and after the onset of a concert event, i.e., the posting rate at the start (cusp) of a song, relative to whatever was happening just before.

## 4 Results: Tweet timing

The following section graphically depicts rates over time from the first Sowoozoo concert while reporting statistical assessments calculated across all four concerts at once.

Figure 1 shows the smoothed measures of Twitter activity through the Hashtag sample for the first Muster concert. Before the show, there was an average of 200–500 original and Retweet Tweets per minutes using the hashtag #Sowoozoo with Retweets picking up after the first live music set and cresting several times over the course of the show. Through observation, we find that Retweets drop or plateau through the Live music segments (starred purple) and rise during most instances of talking, video clips, and when BTS was off stage. The estimated Likes rate varies from 1 to 3 times the Retweet rate, with proportions becoming more steep during the non-musical segments as well.

**Figure 2:**
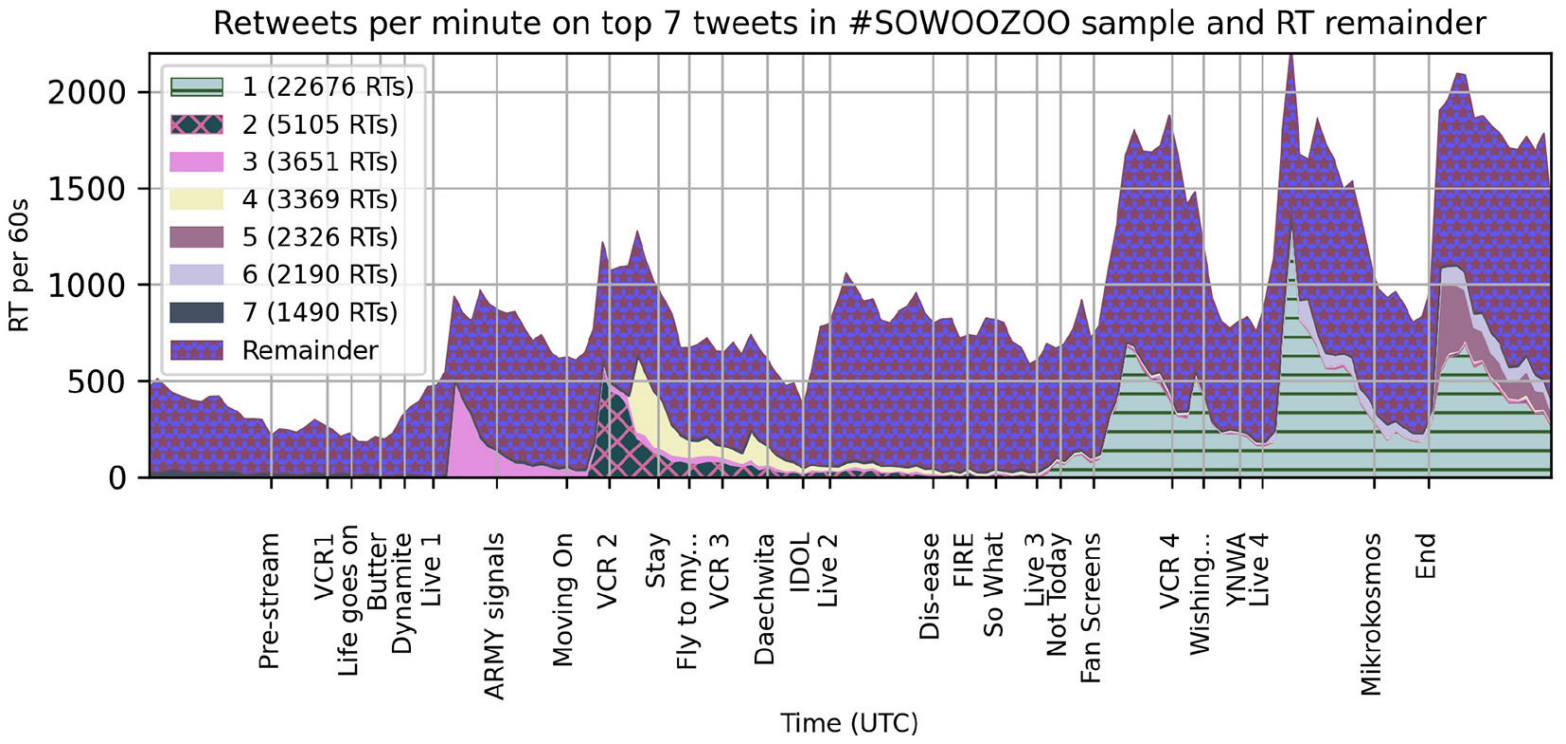
Stacked Retweets rates of the seven most retweeted Tweets in #Sowoozoo during the Day 1 concert under the remainder of Retweets, counted in retweet posts per minute. X-axis corresponds to timeline in Figure 1.

Popular Tweets strongly influence the shape of Hashtag sampled datasets with peaks in Retweets over time usually driven by individual hypervisible posts. To check the impact of popular Tweets during these concerts, Figure 2 shows the number of Retweets per minute attributable to each of the top seven Tweets in this data set. Retweets of these posts constitute 28% of all hashtagged Retweets during the displayed measurement interval.

The typical shape for a Retweet over time is a sharp spike on posting or Retweeting by a large account and then a gradual tapering off. Some of these top Tweets, say 2 and 3 in Figure 2, follow that structure with a small spike also visible in the total Retweet rate. However, the most popular Tweet in the set, shown in horizontally stripped mint green, goes up and down with the concert program events, suggesting that contour of Retweets is varying more with attention to the time line than to just the presence of retweetable content. These valuable Tweets are in users' timelines, but at some moments in the show, fewer users are looking.

By the Likes and Retweet rates, it seems concert content had a strong impact on users engagement on this platform with the online audience focusing more on the broadcast while music was being performed. To test this pattern across concerts, we calculate the Rate Shift ratio for each contiguous program segment and tested the factor of concert event type on the rate of Retweets per minute with a Welch ANOVA across all four concerts. This test of means is more reliable for samples of unequal variance and number (Liu, 2015), and finds a significant effect of concert event type, *F*_(3,27.96)_ = 5.69, *p* < 0.005.

**Figure 3:**
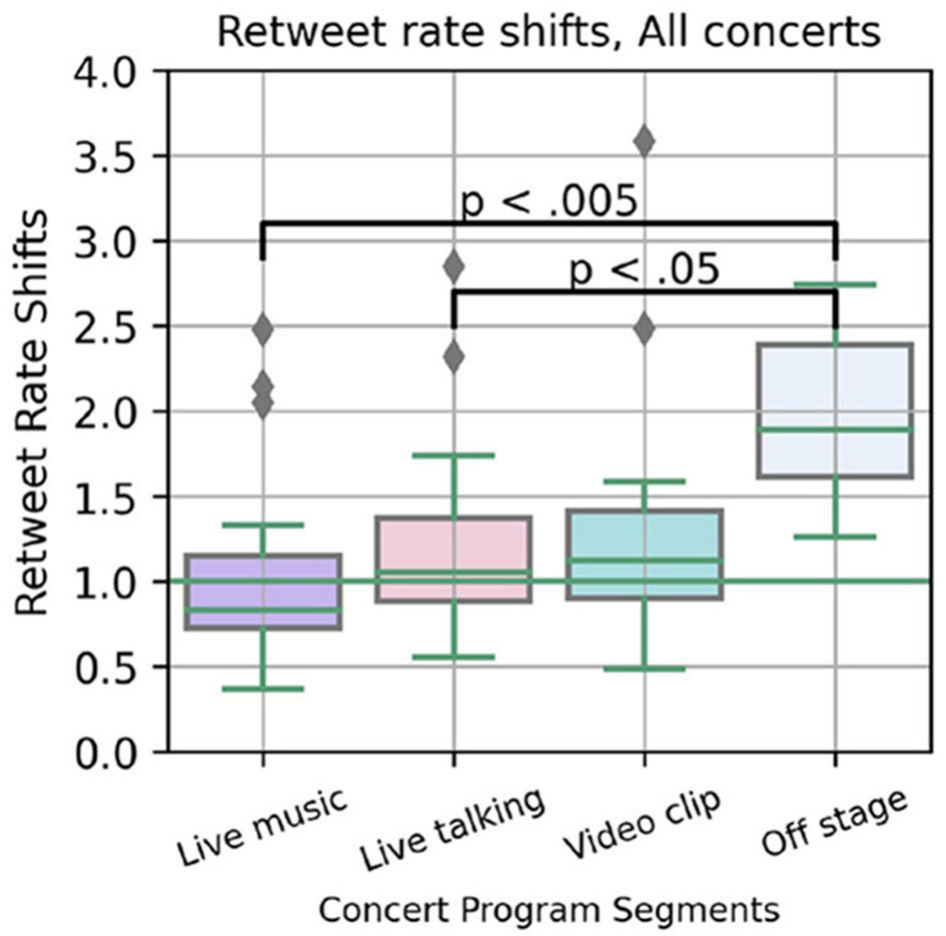
Relative Retweet posting Rate Shift per concert program segment (set of songs, talking interval, etc.), aggregated across four concerts.

Figure 3 plots the distributions of relative Retweet rate per concert event with significant outcomes of the *post-hoc* pairwise comparisons (Games-Howell, again to allow for unequal sample sizes). While there were some exceptions across these four performances, evidence of attention to Twitter decreased when BTS was singing and dancing (median below the green 1.0 threshold), and it increased most dramatically when they were fully off stage or not on the stream.

### 4.1 Original Tweet rates

The pattern of Retweet rates fits with the expected narrative of fans' priorities during a concert performance, however one type of Twitter activity did not follow the same trajectory. In Figure 1, the dark line tracing the Original Tweet rate (Originals) shows that while this activity is much rarer than Retweets, the time series often increases in moments when the other rates drop. Retweet and Original rate time series are mildly negatively correlated in this first concert (*r* = −0.36). Why would Original Tweets increase at those moments when attention to the timeline seems to go down?

**Figure 4:**
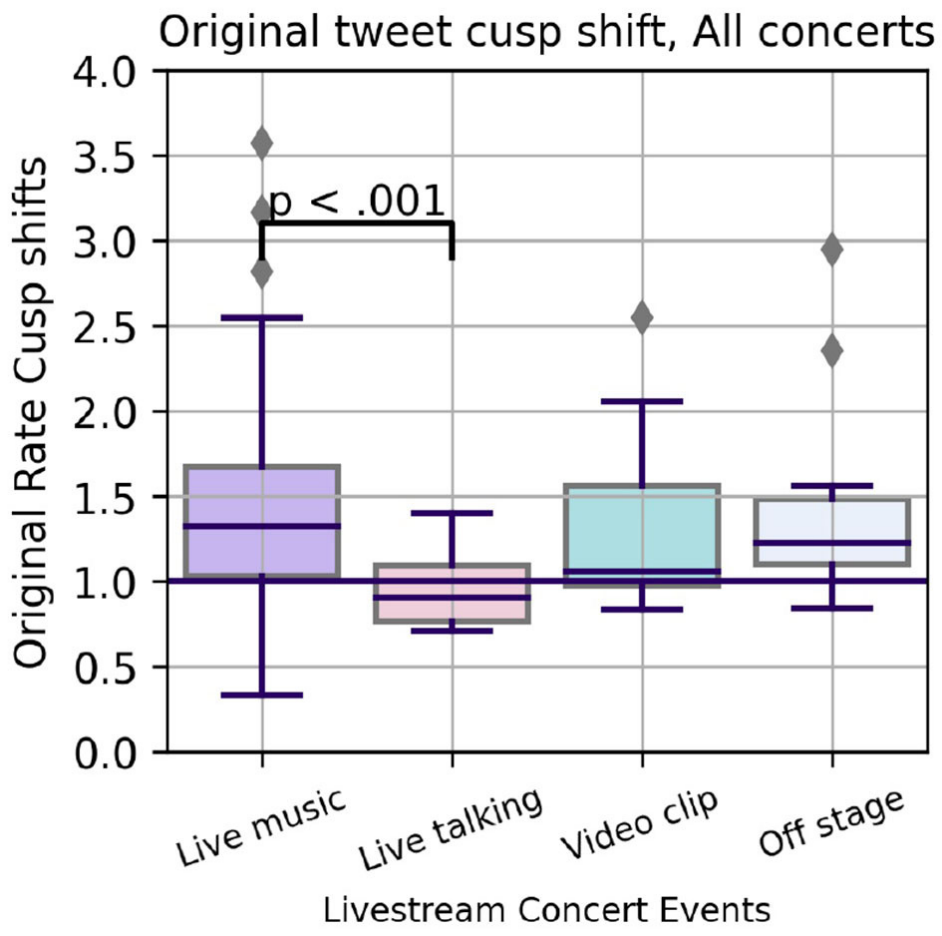
Change in relative posting rate of Original Tweets at the Cusp of program events (individual songs, videos, etc.).

Closer inspection of this pattern suggested short-lived peaks in posting rates at the onset of songs. To check if this pattern was common across concerts, we defined the Cusp Shift ratio, checking for changes in the rate of Original Tweets posted in the minute before and after the start of individual concert program events. A Welch one factor ANOVA finds a significant effect of concert event type on the Cusp Shift, *F*_(3,35.21)_ = 10.04, *p* < 0.0001, with a *post-hoc* test identifying the contrast between the start of Live Music segments (individual songs) vs. intervals of Live Talking. Across these performances and sampling methods, there is a measurable tendency for fans to be suddenly posting more (median of 1.3 or 30% more) at the start of songs while the Original Tweet rate tends to slow at the start of an interval of talking (median < 1.0, below the purple 1.0 threshold in Figure 4).

**Figure 5:**
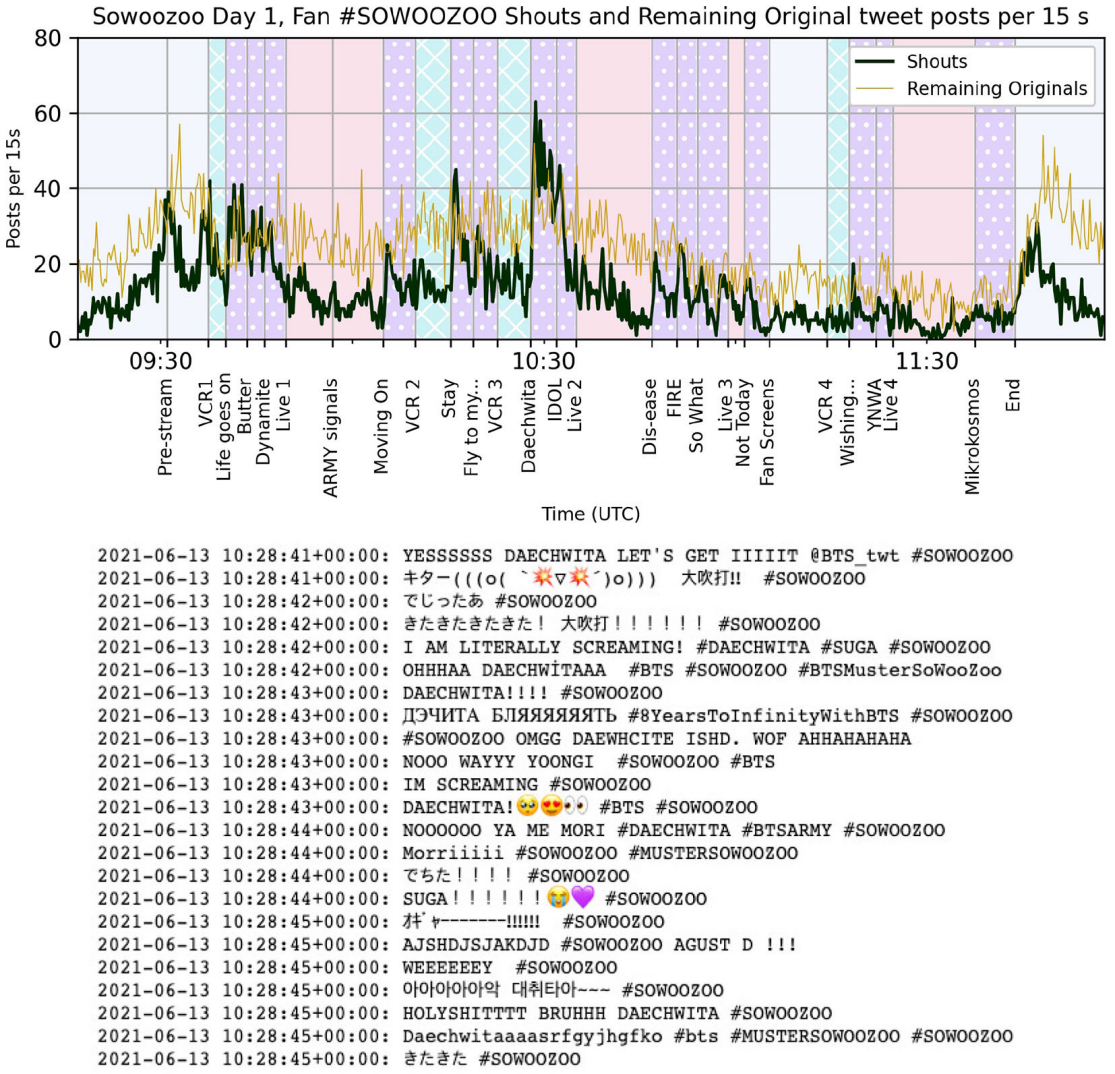
**(Top)** Number of Shout Tweets (Shouts) and the remainder of Original Tweets with the #Sowoozoo hashtag per 15 s interval over the course of the Sowoozoo concert live stream on June 13th, 2021. Background colors mark concert stream content categories as in Figure 1. **(Bottom)** Example of Shout Tweet content, consecutive run excerpt at the start of Daechwita feat. BTS, by Agust D (Suga).

Checking a few instances of these song-triggered spurts of Original Tweets showed a great concentration of short Tweets stating the titles of songs with little more than a hashtag and some intensifying cues, see examples in Figure 5. Short Tweets in this style can be posted without much effort or even a glance at what else might be happening on Twitter. To test if this kind of post was a common, we defined a subcategory of Original Tweet, called *Shout Tweets*, that are original status updates without embedded media and under 60 characters. While many are even shorter, the threshold of 60 characters was chosen to be inclusive across writing systems and allow for concert hashtags. Figure 5 shows the rate of these Shout Tweets counted on 15 s intervals with the remaining Original Tweets (longer and/or with images or videos embedded). At this resolution, the shouting is even more dramatically aligned to the onset of songs, and captures fans' excitement for the special all-member BTS performance of Agust D's Daechwita as they continued this “shouting” for the full duration of the piece.

The variation in Shout Tweet rate with concert program events is extremely strong but over much smaller numbers than the previous posting rates, and here we should acknowledge the limits of Hashtag sampling. It is reasonable to expect that only a small portion of concert attendees were determined to type in the concert hashtags with their shouts of excitement. Comparison of content between the two sampling methods, Hashtag and Stream, exposes more clearly what fans were really posting during these broadcasts.

## 5 Materials and methods: Tweet content analysis

To get a picture of what fans were expressing (Original Tweets) and sharing (Retweets) during these live concert streams, we wanted to look more closely what was being posted. However, these sets of Tweets per concert are much too large to explore in depth. We chose instead to extract subsamples of Tweets from the first Sowoozoo performance, the first set collected by Hashtag, and from the first Permission to Dance performance, collected from the previously-defined Stream.

### 5.1 Subsampling concert datasets

A completely random subsample on the full concert datasets would be quite repetitive, as the most commonly retweeted content dominates these samples. Figure 2 demonstrates the concentration of Retweets in the Hashtag sampled datasets, and the ratios of Original Tweets to Retweets are even more extreme in the Stream samples (see Table 1). Individual users experiences of Twitter through their timelines was also more variable than these samplings suggest. While the most popular posts would pop up multiple times in their personal view of platform activity, users often ended up glossing over these repeats (sometimes with the help of algorithmic influences) and paying more attention to Original Posts by their *Mutuals* (people they follow who also follow them). As the majority of Original Posts get zero Retweets, a naive random subsample would miss out on these direct expressions by individual fans.

In order to explore content patterns across this great range of visibility, we used the total number of Retweets recorded during the concert intervals to stratify the unique posts in each sample and picked a few tiers of popularity to draw from. We defined four ranges of Tweets in the first Sowoozoo and first Permission to Dance concerts: the top most retweeted in the dataset (Top RT), Tweets with 6–32 RT (Mid RT), Tweets with 1–3 Retweets (Low RT), and Tweets with no Retweets at the time of sampling (No RT). These strata were then subsampled, randomly for all but the most retweeted, taking 200 from the Sowoozoo sets, and 100 from the PTD on Stage concert sets (with replacement for unrelated or lost content). This stratification also helped compensate for the practical consequences of the two concert sampling methods. Table 2 reports some statistics on these eight subsamples. Descriptors of the subsamples include the size of each strata within each dataset and a rough estimate this strata's visibility based on the follower numbers of the users sharing them. The median statistics point out substantial differences between these datasets with the top Retweets contrasting by an order of magnitude. The differences in median follower counts also suggests that hashtag use increased the visibility of concert-related Tweets beyond many of these users' immediate network.

**Table 2:** Description of database stratification and subsampling used for content analysis with median Tweet statistics and total strata visibility within each sample and across Twitter users' timeline.

**Subsets**		**Visibility**		**Median Tweet statistics**			
**Source**	**Range (selection)**	**Sample**	**Timeline** ^*^	**RT**	**Likes**	**Length**	**Followers**
Hashtag	Top RT (Top 200)	138,607	153,482	718	1,306	172	5,433
Hashtag	Mid RT (Rdm 200)	7,378	44,752	13	38	134	1,199
Hashtag	Low RT (Rdm 200)	3,814	18,988	1	2	127	322
Hashtag	No RT (Rdm 200)	25,230	31,336	0	N/A	71	185
Stream	Top RT (Top 100)	66,848	124,968	7,127	18,050	63	214,783
Stream	Mid RT (Rdm 100)	4,429	24,990	17	37	81	16,531
Stream	Low RT (Rdm 100)	1,410	1,926	1	3	73	3,557
Stream	No RT (Rdm 100)	12,665	12,665	0	N/A	38	498

^*^Indicates estimate from maximum RT counts recorded.

N/A, not available.

### 5.2 Tweet content coding

To develop criteria for content coding, the Top RT and No RT subsamples from the first Sowoozoo concert were iteratively explored for themes that were usefully descriptive and reliably identifiable.

After a first pass to pick out recurring topics, modes of expression, and media use, the emergent codes were reviewed for reliability. Tweets are small dense expressions that depend on a shared cultural context for accurate interpretation, using memes as a shorthand that are easily misinterpreted without specific knowledge. Additionally, these Tweets included text in many languages. The ratio of English was higher in the Stream sample (75%) than the Hashtag sample (44%), but over 30 languages were represented more than 20 times in each concert dataset. Korean, Thai, Japanese, Indonesian, and Spanish commonly occurred in the subsamples pulled for content analysis. Consultation with multiple machine translations and context clues from user profiles and connected Tweets (Replies) were used to interpret individual Tweets across language barriers and unfamiliar references. However, it was still necessary to restrict the codes of this analysis to qualities that could be discerned by the coder around the unresolved ambiguities. An initial set of 35 codes were collapsed to 16 on reviewing the Sowoozoo the Top RT and No RT subsamples. These 16 codes were assessed on the remaining subsamples Tweets. Of these, 12 codes varied substantially in frequency across the subsamples and are reported on below.

More traditional strategies for large text datasets such as affect analysis were not used because of the limits of translation and the medium-typical use of negative language to express positive or ambiguous reactions. The coding process found almost no Tweets that were commenting critically on the concerts beyond the occasional complaint about stream video quality. While shades of admiration would be interesting to classify more finely along the lines of familiar affection, thirst (lust), respect and the like, these Tweets did not always include enough context to reliably distinguish between these modes of appreciation.

The thematic content codes fell into two categories: *topics* as the discernible subject of the post and *tone* pertaining to how these topics were addressed. The thematic content codes were defined as follows:

- **Affection** (tone): Tweets that expressed admiration, gratitude, appreciation, and love in text or with related emoji such as 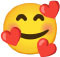, 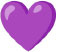, 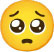 in appropriate contexts.
- **Intensity** (tone): Tweets that used cues typical for these social media spaces to demonstrate intense feelings such as ALLCAPS, repeated characters (with suitable associations), negative emojis (e.g., 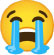 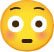 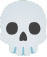), swearing, dramatic punctuation, and accusatory or admonishing language like “HOW DARE” to express a positive sentiment such as admiration or enjoyment.
- **Music** (topic): Tweets that made explicit reference to a piece of music performed during the concert or related to an aspect of musical performance in the show such as dance choreography, voice quality, or rap delivery.
- **Show** (topic): Tweets that addressed the staging, costuming, programming, and more technical aspects of the concert production, including the prerecorded VCRs.
- **Info** (topic): Tweets that relayed specific information about the concert such as the set list, access options to live streams, and translations of band members' speeches.
- **Members** (topic): Tweets about the members of BTS, identified individually or as a group, either by explicit mention or through excerpts of the concert such as edited stills, gifs, and videos with a discernible focus.
- **ARMY** (topic): Tweets that included references to the broader BTS fandom, either through direct mention of ARMY or by speaking in the collective voice such as “We were well fed today.”
- **Self** (topic): Tweets that referred directly to the person posting through first person pronouns and/or verbal descriptions of their experience and reactions to what they were watching.

A few Tweet features were evaluated more systematically: the inclusion of **Media** as identified by hyperlinks, use of the most prominent concert **Hashtag**, mention of the performers' twitter account (**@BTS**), and Tweet length. The additional tone category of Shout Tweet (**Shouts**) was constructed by Tweet length and absence of media, as a test of the pattern found in the posting rate analysis. Media embeddings were additionally assess for whether they featured material from the performance, **Concert Video** and **Concert Stills**, when this could be verified.

A last note on coding challenges: as some of this analysis was performed many months after the performances, it was not possible the verify the content or even the type of media embedded in some of the sampled Tweets. The distribution of copyrighted materials often lead to Tweets and accounts getting suspended, resulting in entire Tweets disappearing or becoming partially obscured when quoted Tweets were deleted. If the subject of the absent media could not be inferred from context, the Tweet was dropped from the subsample and replaced.

Given the many constraints and complications in how the codes were developed and applied, the analysis that follows is principally descriptive with a focus on large scale trends.

## 6 Results: Tweet content analysis

The following section discusses patterns in the content identified in subsamples of Tweets taken from the Hashtag sampled Sowoozoo Day 1 concert Tweet dataset and the Kpop Stream sampled Permission to Dance on Stage Tweet dataset. With samplings stratified by Tweet popularity (Number of Retweets), some trends are similar between the two concert sets and others are noticeably different.

**Figure 6:**
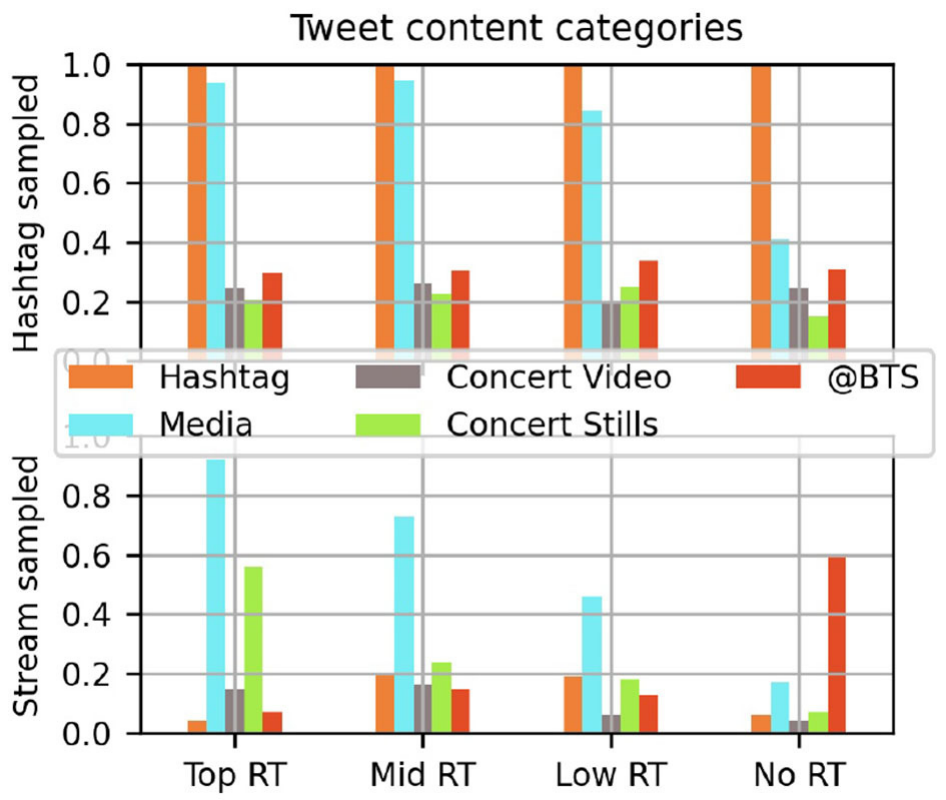
Frequencies of Tweet content categories in subsamples by popularity based on Retweets (RT) and sample source.

**Figure 7:**
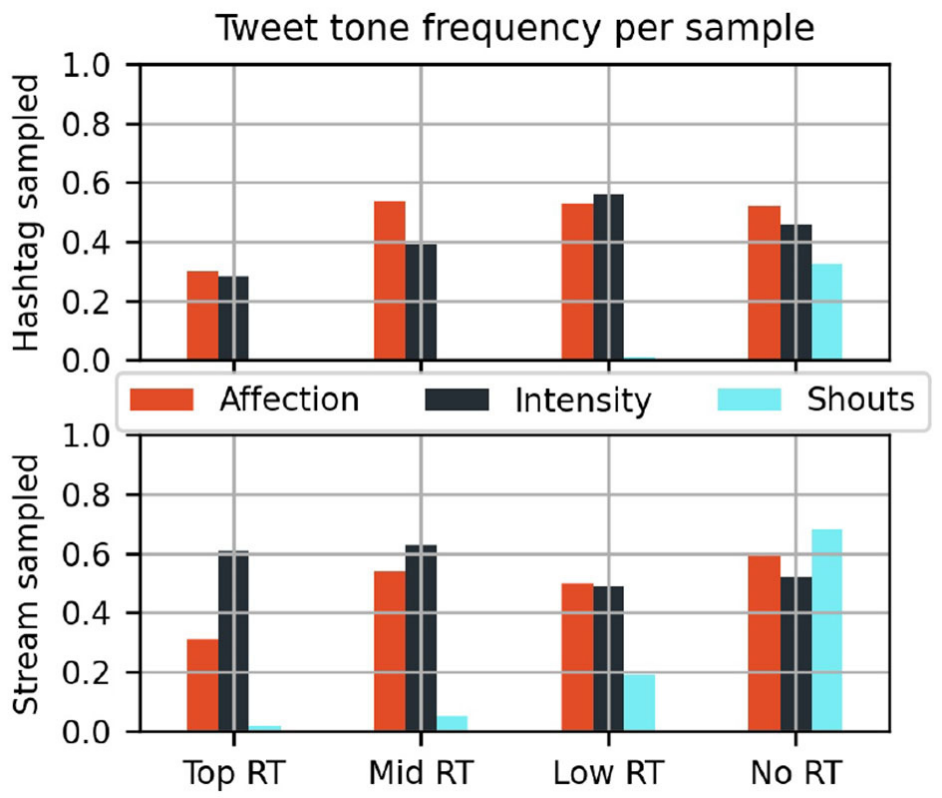
Frequency of Tweet message tones in subsamples by popularity and sample source.

The bar graphs in Figures 6, 7 report the frequency of Tweet content categories and tones across the subsamples. Tweets from the first concert dataset evidently all included the concert hashtag, while the use of #PTD_ON_STAGE hashtag was rare in the Stream sampled subset, at most 20% in each range of popularity. Another contrast between these samplings is the inclusion of the performers' username. At all levels, BTS were @ed in roughly a quarter of Tweets using the #Sowoozoo hashtag, often with a slew of popular hashtags appended to the main text. During PTD on Stage, the more widely circulated Tweets caught in the Stream sample rarely mentioned this official account, however it was often in Tweets that were never retweeted.

Figure 6 also shows a strong trend by popularity across the two samplings: the prominence of embedded media in more popular posts. Less than 10% of either concert's top Retweets were all text, and the proportion drops across tiers of popularity. Maybe by design, the Hashtag sampled Tweets had higher rates of media inclusion at all levels of popularity. The particular value of different media types is harder to assess as so many had been taken down by the time of assessment. That surviving stills were substantially more popular than video in the later Stream sample may have been another strategic choice after fans saw their retweeted material disappear from previous concerts.

The tone of Tweets show distinct trends by popularity and sampling method. Direct expressions of affection were less common in the most widely shared posts (red bars of Figure 7), however intensity cues differed by popularity only in the Hashtag sample. Posts do not need to be staid in their expression to be popular, fandom-typical superlatives do not stop materials from being shared, however those who are posting may be strategic in choosing a more respectable tone for messages tagged for external visibility.

Included with the tone codes is also the frequency of Shout Tweets. These short and media-less Tweets rarely get to circulate after an initial posting, and they are more common in the Stream sample. This is consistent with the assumption that fans cheering online this way are more interested in expressing themselves in the moment than having these messages be heard by an official ear.

**Figure 8:**
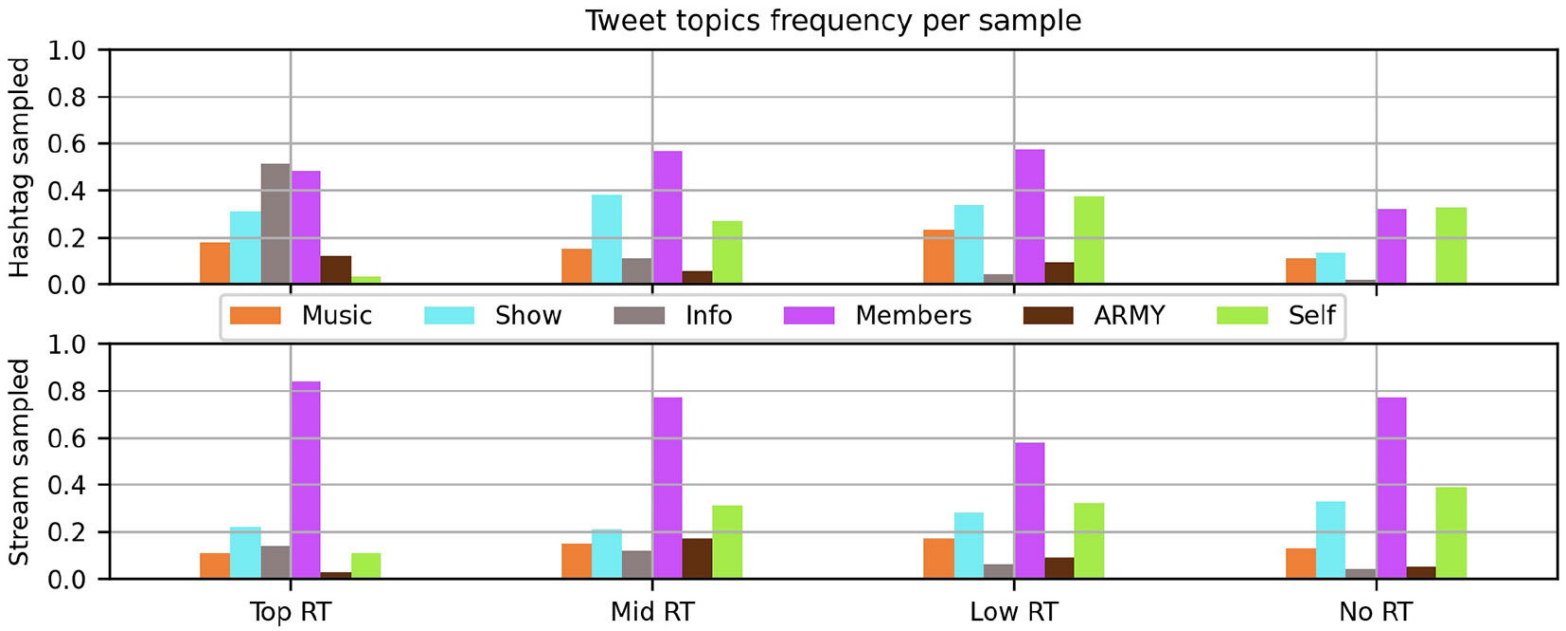
Frequency of coded topics per subsample, by popularity and sample source.

Figure 8 shows the relative frequency of topics with two prominent distinctions by popularity and one obvious difference between samplings. Around half of the most popular Tweets (Top RT) within the Hashtag sample are sharing practical information about the concert. Carrying privileged details like set lists with accurate track names and translations that could differ or be missing from the broadcast, these widely shared posts seemed to keep to the facts without affectionate or intensity tone markers. Such examples of fan labor are intentionally given hashtags to facilitate strangers finding the posts and making use of the work.

Tweets that mention the user posting (Self) shift in both samples by popularity. While there are a few widely Retweeted posts that use phrases such as “I am thankful,” the posts in the low to no Retweet range are much more likely to explicitly mention the user's own feelings and experiences. This could be a consequence of their networks: it is much less costly to share personal feelings on accounts with a smaller numbers of followers (medians in Table 2). At the same time, explicit mentions of ARMY or even vague communal terms for the attending audience were not particularly common under any circumstances.

The principle difference in topic between the two samplings was how often BTS band members were the subject of Tweets in the Stream sample. At the higher levels of popularity, the majority of Tweets in this sample featured concert stills or video with a focus on individual members. Given the frequent use of Intensity tone cues, Tweets of shocked admiration, gregarious thirst, and overwhelming adoration for BTS dominated the popular range of the Stream sample. These forms of appreciative content were typical on Kpop Twitter, however they were generally not intended to be seen by the people being described. This distinction between sampling methods underlines how many of the Tweets and Retweets accompanying these concerts are materials and messages exclusively for other fans. In contrast, Tweets in the Stream sample that seemed instead addressed to members, text starting with BTS's username, did not get retweeted at all.

While the posting rates and interaction timing made clear that these Twitter users tended to be very interested in the music performances of these live streamed concerts, it was not so frequently a topic of discussion, between 10 and 22% of each subset. Slightly more common were comments related to production choices (shown in Figure 8) particularly in the Retweeted hashtagged subsamples.

The most surprising absence from the content of these Tweets were mentions of the pandemic lockdown conditions that initially made live streamed concerts necessary. The first round of coding on half the Sowoozoo subsamples did not identify themes related to COVID-19 pandemic, performance restrictions, or the like because it never came up. A second search through the full databases of Tweet texts using a few keywords (virtual, pandemic, COVID, lockdown, and restriction) found only around 1,000 Tweets across posts during all concerts. The highest concentration were from Permission to Dance in LA, a show that had a face-to-face audience as well as those attending the live stream. About 0.5% of that concert's dataset included these keywords, wherein the majority were direct quotes of BTS member RM stating “Fuck COVID!” on stage.

## 7 Discussion

Much of the research on remote audiences has used concerts and audiences of a very different character than observed here: genres of music that rarely call for audience interaction, free concerts consumed more casually than an expensive in-person shows, new and unfamiliar broadcasting technologies, and small artificially constructed audiences. Here, instead, is captured unprompted activity from an extremely large (100, 000) paying audience attending highly anticipated performances for a community with a strong culture of active engagement during performances, extensive experience with live streaming, and access to an established networked space for live and near-synchronous fan-fan interaction. This dataset shows how a resourceful audience can extend and enrich their live stream concert experience with actions not supported by existing broadcasting platforms. Taken in combination, patterns in this distinct collection of fan Tweets during live streamed concerts have implications for how we understand what audience members add to concert performances, their own experiences and each others', and why so many went outside of the live stream platform to act and interact.

### 7.1 The audience's interest in the audience

For many fans of BTS, live stream concerts are as close as they will ever come to seeing the group perform live. Around the world, fans bought tickets and arranged their schedules to watch the concerts, some waking before dawn for the chance to see these seven on stage. And yet the rates of Likes and Retweets reported in our analysis of Tweet Timing demonstrate that many audience members were checking their Timelines during these broadcasts. Given the apparent value of these performances, why would so many be turning their attention away from the concert live stream to look at social media?

Social connectedness is an important motivation for attending concerts face-to-face (Kjus and Danielsen, 2014; Brown and Knox, 2017). The livechats on many broadcasting platforms allow some of this mutual awareness, a narrow channel over which everyone can shout to each other. When chat postings are in the hundreds, say Facebook Live streams of classical music concerts, participants can feel empowered by the chance to express their experience (Nguyen, 2018), however interactions are few and far between (Vandenberg and Berghman, 2023). On platforms with more active livechat practices, receiving direct acknowledgment of a comment is even more technically difficult because of the rapid rate of postings, and exchanges with people they know off-stream seem to be more social rewarding than responses from strangers (Vandenberg, 2022). And while people who comment frequently and in detail are more likely to feel like their feelings are shared with other live stream audience members (secondary analysis, Swarbrick, 2021), those willing to be so active are relatively few (1 in 5). Audience-audience interaction over live stream livechats seems more limited and onerous than many concertgoers want.

Already a proven point of access to both BTS's ARMY mutuals and interested strangers (Park et al., 2021a), Twitter offered these audience members an interesting alternative space for chat-like actions. Instead of dropping their comments in a churning live stream chatroom, thousands of audience members opted to prioritize “interpersonal communication” (Kjus and Danielsen, 2014) by shared Tweets where these would be shown to people they know. Whether in realtime or some minutes later, Twitter's array of engagement options then granted their followers a chance to acknowledge such Tweets directly with as little effort as clicking a heart icon or as much as writing a reply. Though we cannot say for certain what exactly each Like or Retweet action might mean (Park et al., 2021b), these engagements confirmed that someone else had seen the user's post and wanted the user to know it. Like eye-contact in a crowd, such light direct interactions may foster *ittaikan* between remote audience members (Tarumi et al., 2017).

The timing of Twitter engagement speaks to a negotiation of attention between what was happening on stage, sharing their own experiences (Original Tweet posting rates), and tracking what was happening in the audience (Retweet rates and estimated Like rates). There were inevitable time lags between posting a Tweet and seeing engagement from other users, particularly when the music called users' attention back to the concert live stream. However, asynchrony was always part of this social media platform. With an interface that only jumped to the latest material upon request, users were familiar with interpreting material presented as from the (recent) past. In contrast to busy livechats, the window of opportunity for engagement on any given Tweet extended for minutes, hours, even days after posting. This persistence of Tweets allows shared feeling and social connectedness between audience members to be reinforced well after the concert. The burst Twitter activity after the end of the concert stream looks a lot like the excited conversations between seat neighbors after an in-person show, even when much of the content being passed around was posted earlier, like in Figure 2.

The varied use of hashtags across these performances highlights these concert attendees' complex communication goals. In a livechat, any message would be broadcast to all watching the chat and potentially retained by the broadcasting platform. On a platform like Twitter, these online concert attendees could at least partially control the visibility of their comments. If they were only interested in the performers, their engagement would have been entirely focused on official accounts. Instead, filtering out Retweets of and Replies to official accounts only decreased these samplings by at most 20% of posts and 25% of unique users. Socially connected fans could also coordinate to share their concert excitement over more private channels. The public Twitter activity studied in this paper is the result of fans choosing to commune with a more manageable number of friends and strangers, as they would at a face-to-face show.

Still, the content posted can be intended for specific segments of the audience. Small accounts might use a hashtag to find wider engagement and build on their online community. Large accounts may instead be more selective of which posts carry hashtags, as these also invite scrutiny, both to the legality of the material being shared and to the tone and topics of discussion. Topics and tones of content that fans want to exchange with likeminded users without the risk of exposure to the performers or judgement by unsympathetic strangers. In-person, these reactions can be shared in the moment without permanent records. Online, fans still manage with the help of mitigating strategies like specialized terminology and the avoidance of keywords.

### 7.2 Audiences online vs. in-person

One of the most widely used tools for audiences at Kpop concerts is the camera phone. Fans use these both like opera glasses to zoom in and focus on action on a big stage and like cameras to preserve these precious moments from a “first-person aesthetic” (Glitsos, 2018), fulfilling the desire of a “possessive spectator” (Mulvey, 2006) with clips to be revisited. In an online broadcast, Retweets of extracted stream footage function very similarly. The work of capturing and processing live stream video takes technical resources and skills well beyond hitting a phone's record button. However, reblogging excerpts from fans capable of performing this work is easy and yields very similar results: a catalog of favorite moments to be reviewed and shared at will.

The Retweeting of information, like translations and set lists, is much like how audience members turn to their seat neighbors to ask what is happening and passing the news onto their accompanying friends. This kind of mutual support is especially important in multilingual and multi-cultural communities like Kpop fandom. A substantial amount of teaching is needed to help *baby ARMYs* (BTS fans who are new to the fandom) adapt to the extensive traditions of this subculture.

Of course, audiences at these shows are also accustomed to expressing intense excitement and joy, and the Shout Tweets demonstrate how strongly they desire to share these feelings, jumping to secondary platforms to scream as best they can with their followers and (using hashtags) with strangers too. Doing so on Twitter enabled their virtual screams to be “heard” (Charron, 2017) during and after the fact. Given the high flow of postings through these concerts, some Shout Tweets may pass without a chance to be liked. However, the resulting wall of Tweets shouting the same song title is easy to recognize as a parallel to the screams of fans at in-person concerts to the opening bars of favorite tracks (and to the collective rush of livechat crowdspeak.)

This virtual shouting also aligned with the patterns of behavior observed on the social music platform SoundCloud. A qualitative analysis of comments left by users on individual tracks, Hubbles et al. (2017) found these to be primarily short and positive toward the song or the artist while the use of Reply function was minimal. They characterized the purpose of such comments as a potential stand-in for mildly interactive experiences, creating a sense of social presence even without direct synchronous interaction (Ducheneaut et al., 2006). Posting on persistent platforms can also satisfy fans desire to demonstrate their worship of the artists, like being seen at the venue and sharing photos after the fact (Brown and Knox, 2017).

While this study cannot address the subjective experience of audience members during these live-stream performances, their observed activity suggests a great potential for rewarding expression and direct mutual recognition with friends and strangers, with just a little more asynchrony than is possible face-to-face.

### 7.3 Beyond Livechat

On live streaming platforms like Twitch.TV, or Kpop-oriented VLive (now part of Weverse), most of the strategies to coordinate commenting behavior are structured around communications from the audience to the performer (Jodén and Strandell, 2022). Topics are encouraged with repetition and the success of the communication is marked by a change of content in the live stream. During these concerts, as with other highly-produced live streamed concerts (Rendell, 2021), the performers being live streamed were not attending to the livechat in realtime. Other than a quick greeting before or after the show, there was no suggestion of attention from the performers to the audience's activity in this space. As such, the chat was left for the audience to interact with each other, without the coordinating influence of performer feedback or the cultivated shared culture built around popular streamer channels. Some of the audience used this broadcast platform feature during the performance, and it may have satisfied their need to express themselves, be seen, and feel like their experience was shared with a larger group of like-minded people. However, the technical impediments to social connection on this medium are numerous. The audience members' activity observed on Twitter during these shows suggests that a substantial number of saw a benefit to moving their interactions to a more hospitable space.

Besides offering convenient direct near- or a-synchronous interaction with mutuals and strangers, Twitter as a platform allowed for richer materials to be posted than a livechat. Much of what was most widely shared fit with the content tweeted from face-to-face shows (Bennett, 2014; Kjus and Danielsen, 2014): images and videoclips of the performances, privileged information like setlists, as well as more personal and affective reactions to the events. As is common for live-tweeted events, the number of accounts making original posts was much smaller than the network of users Retweeting and Liking what was share. However, unlike live Tweets from most face-to-face music performances, Twitter engagements appears to have been coming from users who were also attending the show. By propagating concert material and related fan commentary in a public networked social space, these twitter users are satisfying a branch of common concert audience activities unsupported by livechat.

While concert feed material like video excerpts were highly prized during and after these concerts, many of these Tweets were soon removed and some posting users suspended for uploading copyrighted material. For those capturing and sharing live stream concert footage, a sanctioned mechanism or channel could alleviate concerns around illicit distribution and heighten their sense of ownership and participation in the concert. Additionally, those who are only able to attend online (or “offline” after the performance) may get a more immersive experience from the first-person perspectives and feel more embedded in the audience. As many BTS fans will come for the realtime performance experience either way, even watching together the same tour show over multiple rebroadcasts, we question the financial risk of letting fans hold onto their favorite moments with personal clips.

The range of audience-to-audience interactions within broadcast platforms could be expanded in many ways, from recommending moments to friends and fans to sharing reactions big and small. Fans want to hear each other cheer and to feel heard by having their experiences acknowledged and mirrored in the people around them. This two-way connectivity needs to be fast, negotiable, and of a manageable scale. Fans want to choose when to attend to the stage or when to attend to their neighbors with a chance for low effort mutual recognition. Allowing concert attendees to find each other in the virtual crowd, attenuating the “roar” of the crowd, and to “capture” concert moments could greatly improve the audience experience. As this research shows, if the audience doesn't like the options for interaction within a broadcasting platform, they can and will seek it elsewhere.

## 8 Conclusions

Online concerts were embraced by many artists during the initial COVID-19 pandemic response, and fans around the world have tuned into these to see their favorite artists perform in realtime. Through analyses of Twitter data across four live stream BTS concerts, we found audiences members acting and interacting online throughout the shows. These fans leveraged their existing social networks on Twitter to recreate many common in-person audience behaviors such as posting Shout Tweets to cheer at the start of songs and capturing records of their favorite concert moments. Audience members shifted their attention between the concert streams and their curated view of other audience members reactions with less Twitter activity during music sets and big increases in posting activity when BTS was off stage. We also observed distinct patterns in what content audience members shared with a priority to Tweets carrying high quality information (translations and set lists) and edited video and stills from the concert feed. Lastly, these concert attendees were strategic in how they used the hashtags, dropping easily-searched markers from Tweets with content intended only for other fans to see.

This audience reconstructed a concert experience denied to them by geography and a pandemic by finding each other outside of the official online venue. Twitter, now X.com, may not always function as a reliable platform for this community, however it served to demonstrate how live stream concerts can still satisfy audiences' interest in expressions of excitement, mutual recognition, the capture of precious memories, and extending their celebration of collective fannish devotion well past the end of the broadcast.

## Data availability statement

Reduced versions of the datasets presented in this study can be found in an online repository and with the open analysis Github repository: https://doi.org/10.6084/m9.figshare.24260452, https://github.com/finn42/Concert_Twt_Open/.

## Ethics statement

Ethical approval was not required for the studies involving humans because this public social media activity was collected through licensed use of the Twitter Streaming API, in accordance with the platform's terms of service. The studies were conducted in accordance with the local legislation and institutional requirements. Written informed consent for participation was not required from the participants or the participants' legal guardians/next of kin in accordance with the national legislation and institutional requirements because this form of data collection was consented to in the user agreements. Additional steps to de-identify user accounts were taken before reporting and public release, further reducing risk to participants.

## Author contributions

FU researched the literature, performed the analyses after data collection, and wrote the first draft of the manuscript. JL supported the data collection, research the literature, and contributed to the Sections 2 and 7 of the paper. SP researched the literature and contributed to the Sections 2 and 7 of the paper. FU, JL, and SP collaborated on the conceptualization of this study, edited the manuscript, and approved the final version of the manuscript. All authors contributed to the article and approved the submitted version.
