# Supplementary material for: Audience reconstructed: social media interaction by BTS fans during live stream concerts
Source: Front Psychol. 2024 Apr 18;15:1214930. doi: 10.3389/fpsyg.2024.1214930 (PMC11066592; doi:10.3389/fpsyg.2024.1214930)
Supplement: Supplementary file 1 [file Data_Sheet_1.PDF]

# Supplementary Material

## 1 PUBLISHED TWEET DATA SETS

By the licensing agreement and standard practices of the Centre of an Informed Public, the complete datasets collected via the Twitter API can not be published in their original form. The following section details how these data have been prepared for open release with filtering and depersonalisation. Interested parties may be also contact the authors to negotiate usage of the original set under the terms set by the University of Washington's Center for an Informed Public and its licensing agreement with the company formally known as Twitter.

To satisfy these constraints and protect sampled population of users, we employed specific strategies in both reporting on tweet content and reducing the datasets for the sake of transparency. The process to depersonalise these datasets is described herecan be downloaded here.

Firstly, each set of tweets was filtered for posts from users with warding notices in their bios along the lines of "DONT USE FOR X". Common terms against citation were searched for in English and Korean in the field of user descriptions. This effort identified 12 users requesting exclusion and filtering out their content removed a few hundred tweets total.

Secondly, these datasets were filtered for tweets retweeting or replying to a list of official BTS related accounts, as described in the paper.

As a general strategy, published citation of individual tweets' contents never included individual usernames or user numbers. For the published datasets, the information retained is specific to the scale of data released.

### 1.1 Tweet timing data sets

To transparently report on the analysis of tweet timing, records of each tweet in these filtered datasets are retained, with most identifying information removed.

Six databases of tweets were captured via Hashtag or Kpop Stream: the four concerts studied here and the two recordings of the Kpop Stream a week before and after Permission to Dance On Stage in Seoul. After filtering, each of these set of tweets were reduced by dropping or replacing identifiable information in key fields. The fields of tweet information retained as is were: timing of tweets posting, tweet statistics at time of sample (numbers of likes, retweets, replies and quotes), the posting users' statistics at time of posting (number of followers, following), and retweeted or quote-tweeted or replied-to user statistics if applicable. In each dataset, all fields with the user id numbers and tweet id numbers were replaced using a hashtable, preserving some of the structure of the timeline and network properties without details that could be used to identify individual accounts. Lastly, a set of new fields were added to replace some of the information lost in dropping the original tweet texts: tweet length (number of characters), media inclusion (binary, 1 if included photos media or quoted tweet), and whether the tweet fell into the categories of Origina, Reply, Retweet, and Quote tweet (not exclusive.)

The exact code used to conduct this reduction and exact field names are retrievable via the analytical notebooks linked above. Data is saved as csv files of with names in the form of "fan_Tweets_PTD_LA4_reduced.csv".

### 1.2 Coded content data set

The second set of datasets are the subsamples studied for content themes. For these smaller sets of tweets, tweet ids are retained, the original tweet content (text with original media hyperlinks), tweet and user statistics, and the codes assigned. For the Sowoozoo datasets, these are complete lists of the original 800 tweets coded. For the PTD_ON1 subsamples, we are publishing both the coded tweet set (400 tweets)and the larger set of tweets with records of the tweets replace because they were lost or not concert related (“_with_replaced.csv”).

Personal information such as username or user id, for the poster or the retweeted account, are excluded. However, if the tweet continues to exist on the X.com database, some of these details can be retrieved via the tweet id. For the sake of transparency, given the subjetivity of content coding and the number of tweets that have already disappear from this platform’s historic database, we have opted to supplement minimal id information with the original tweet text.

## 2 KPOP STREAM TWITTER ACTIVITY

In two of the concerts studied, concert hashtags were not used consistently enough to capture substantial numbers of tweets. To get a broader view of fans and audience members activity during these livestreams, we opted to instead capture all tweets in the previously defined Kpop Stream. Besides some keywords, this stream collected tweets from a set of twitter users ($\leq 5000$) quasi-randomly selected from the intersection of followers to some key accounts in the BTS fandom Twitter network. Users were included if their feeds were active and principally devoted to kpop fandom topics. The selection criteria was intended to capture a rich view of conversation within this fan community on twitter through a moderately representative cross-section of the network. Additionally, this stream captured tweets using project related keywords, included some versions of the suggested concert hashtags.

To check whether it was fair to assume most of these tweets captured on the Kpop stream were related to the live broadcast concerts, we evaluated the posting activity caught by these criteria at other similar times. The same four-hour interval was captured one week prior and one week following the Permission to Dance on Stage in Seoul concert, when no live BTS-related events were scheduled. Given the global participation in the audience for these concerts, sampling at the exact same time of the week ensured the most suitable comparison.

Figure S1 shows the rate of tweets (inclusing retweets) posted per minute during the four hour intervals on each of the three weeks, and clearly ther was much more activity on this stream during this concert than at parallel time, from 4 to 100 times the number of posts for any given minute.

The concert day stream does show a few points of near zero postings, around 09:30 and 12:45. These are artifacts of the sampling process. The streaming API captures up to a maximum number of tweets per minute and when the sampling criteria exceeds that rate limit, it breaks the feed. At these moments, posts by official accounts were triggering a flood of retweets on the stream, all repeats of material excluded from this audience-oriented analysis.

## 3 SECONDARY ANALYSIS OF CORONA CONCERT SURVEY DATA

Activity on twitter does not speak directly to the experience of audience members as they engage in expressive behaviours online. However the Corona concert survey asked participants to report

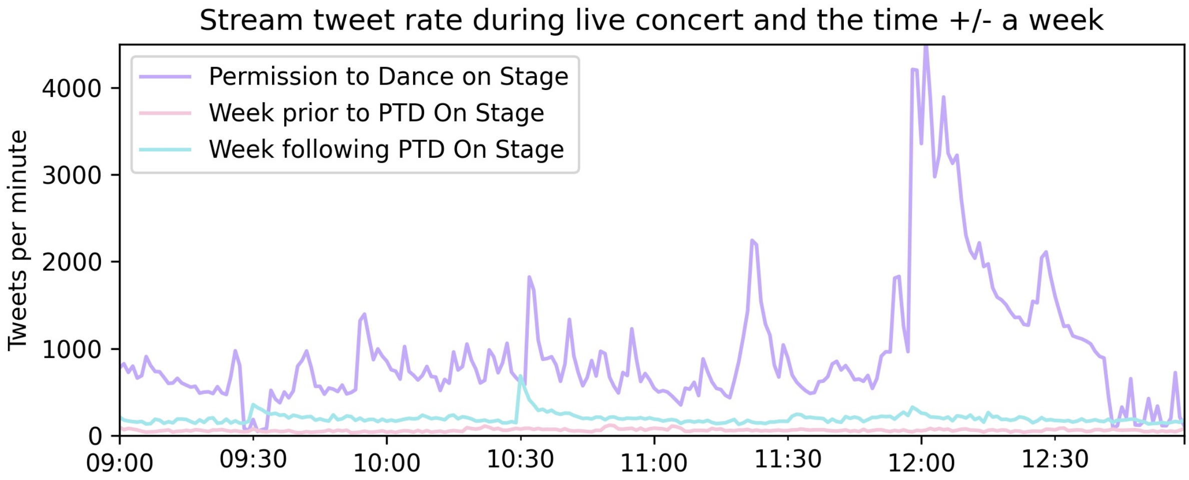


Figure S1. Number of posts per minute captured on the Kpop Stream during the Permission to dance on stage in Seoul concert (2021-10-24 09:00:00-12:59:59 UTC), and at the same time one week prior and following. These counts are generated after filtering out official accounts.

their participatory behaviours at the last remote concert they attended in conjunction with many questions about their experience (Swarbrick et al., 2021). The authors generously shared the anonymisous survey responses on OSF (Swarbrick, 2021), and we reinterpreted the results on a subset of questions after grouping responses by interaction behaviour. We are treating this reinterpretation as exploratory because this dataset has already been subject to thorough statistical modelling. Some of the categories of engagement are quite small, and this survey was performed at a very particular time in the coronavirus pandemic period. Interpretation must respect theses conditions and small numbers.

## 3.1 Remote participation

Participants reported their interaction behaviour by clicking all that applied in the list: “I did not comment at all”, “I clicked on a reaction button (e.g. like)”, “I wrote a brief comment”, “I wrote multiple brief comments”, and “I wrote one or more detailed comments”. Around 354 participants responded to this question and they were grouped by the most effortful action reported:

- No expressive action: only checked “I did not comment at all”, total 91 responses
- Just reaction emoji: checked “I clicked on a reaction button (e.g. like)” and no mention of comments, total 104 responses
- One brief comment: checked only “I wrote a brief comment”, or this and reaction emotion, total 48 responses
- Many brief comments: checked “I wrote multiple brief comments”, total 67 responses
- Detailed comments: checked “I wrote one or more detailed comments”, total 13 responses

As it is not clear what is more effortful, making many brief comments or one or more detailed comments, these last two groups are not exclusive. Only 2 responses of 13 that mentioned making detailed comments did not also report many brief comments.

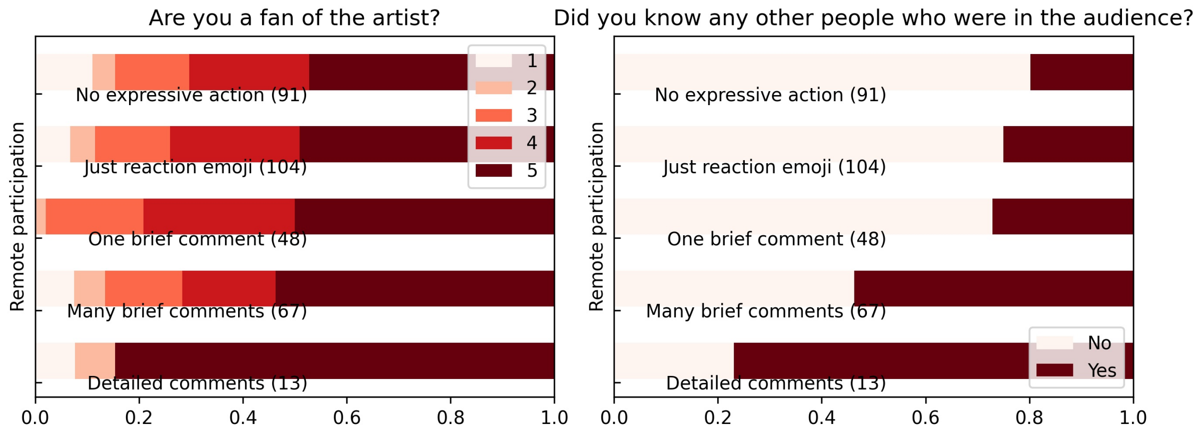


Figure S2. Social context of concert attended, if these respondents were fans of the artist and if they knew other audience members attending remotely, according to how they engaged in remote participation.

## 3.2 Participation and experience

The first notable distinction between remote participation groups is that those that engaged in detailed or frequent comments were more likely to know other people in the remote audience (second plot of Figure S2). Knowing someone did not guarantee more commention activity, however, those who did engage in the broadcast platforms livechats were more likely to be personally aware of other audience members.

In terms of over all experience, shown in Figure S3 those that commented more (in number and details) did show slightly stronger tendency to be captivated or a sense of "being there", particularly in contrast to those who did not perform any expressive actions where other remote attendees could see.

As expected, the biggest difference between these participation categories is in their social experience of the performance, shown in Figure S4. Noticing others, feeling seen, feeling connected and sharing feeling were all more likely to be stronger in those participants who tried to share their experiences through not just one comment, but many. It is interesting to see that just using reaction emoji or making a single comment did shift some of these facets of social connection, but not feeling like others were aware of their presence, or (perhaps as a consequence) feeling connected to other audience members.

While there are many other factors that could be distinguishing these sets are participants, this reanalysis suggests that the action of repeatedly and thoroughly sharing ones' experience is particularly important for feeling seen by other audience members. These expressive actions may be necessary for building that essential sense of unity across the audience watching remotely.

## REFERENCES

[Dataset] Swarbrick, D. (2021). Quarantine concerts: Publication. https://osf.io/skg7h/

Swarbrick, D., Seibt, B., Grinspun, N., and Vuoskoski, J. K. (2021). Corona concerts: The effect of virtual concert characteristics on social connection and kama muta. Frontiers in Psychology 12. doi:10.3389/fpsyg.2021.648448

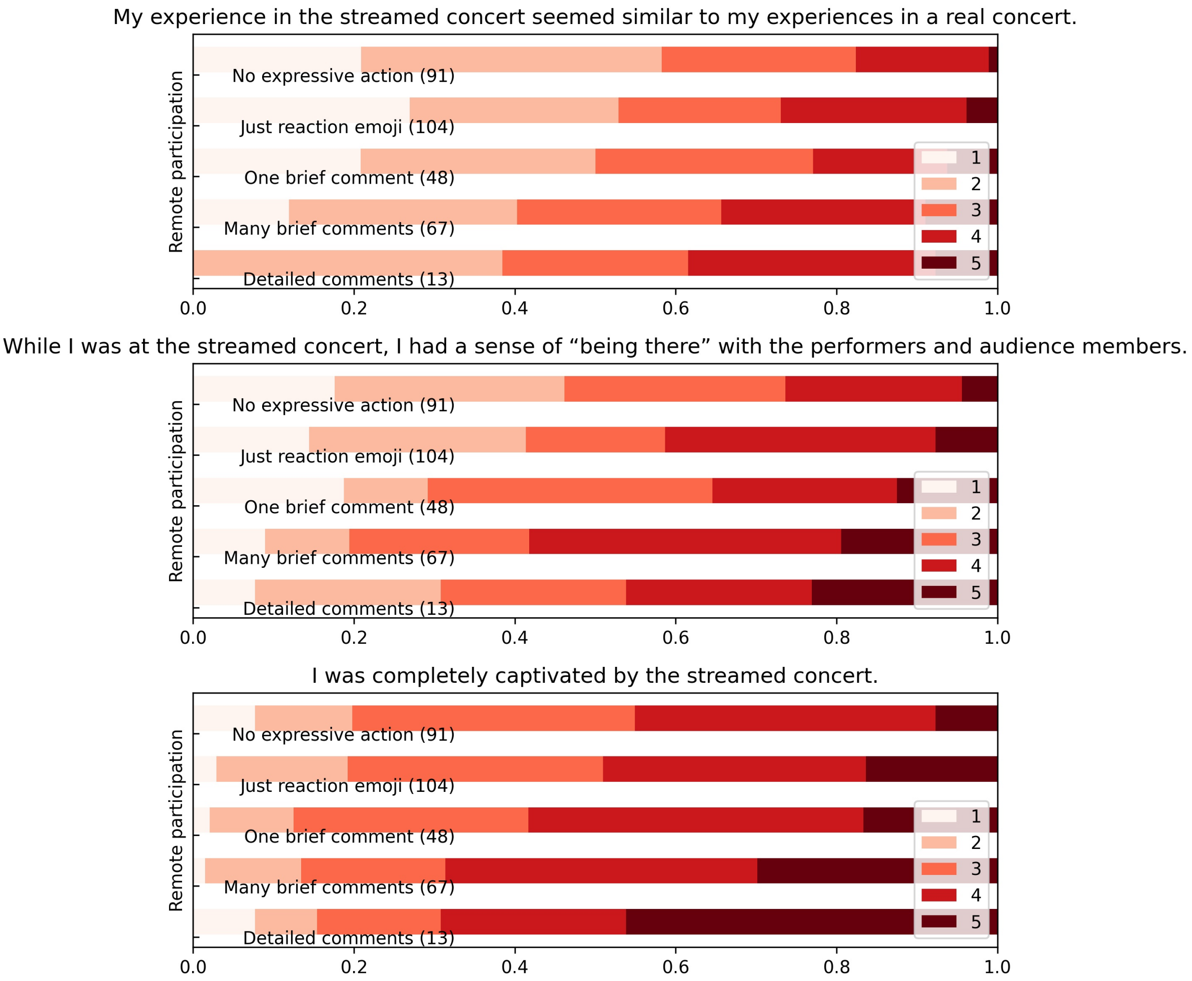


Figure S3. General experience of the performance, according to how these survey respondents engaged in remote participation.

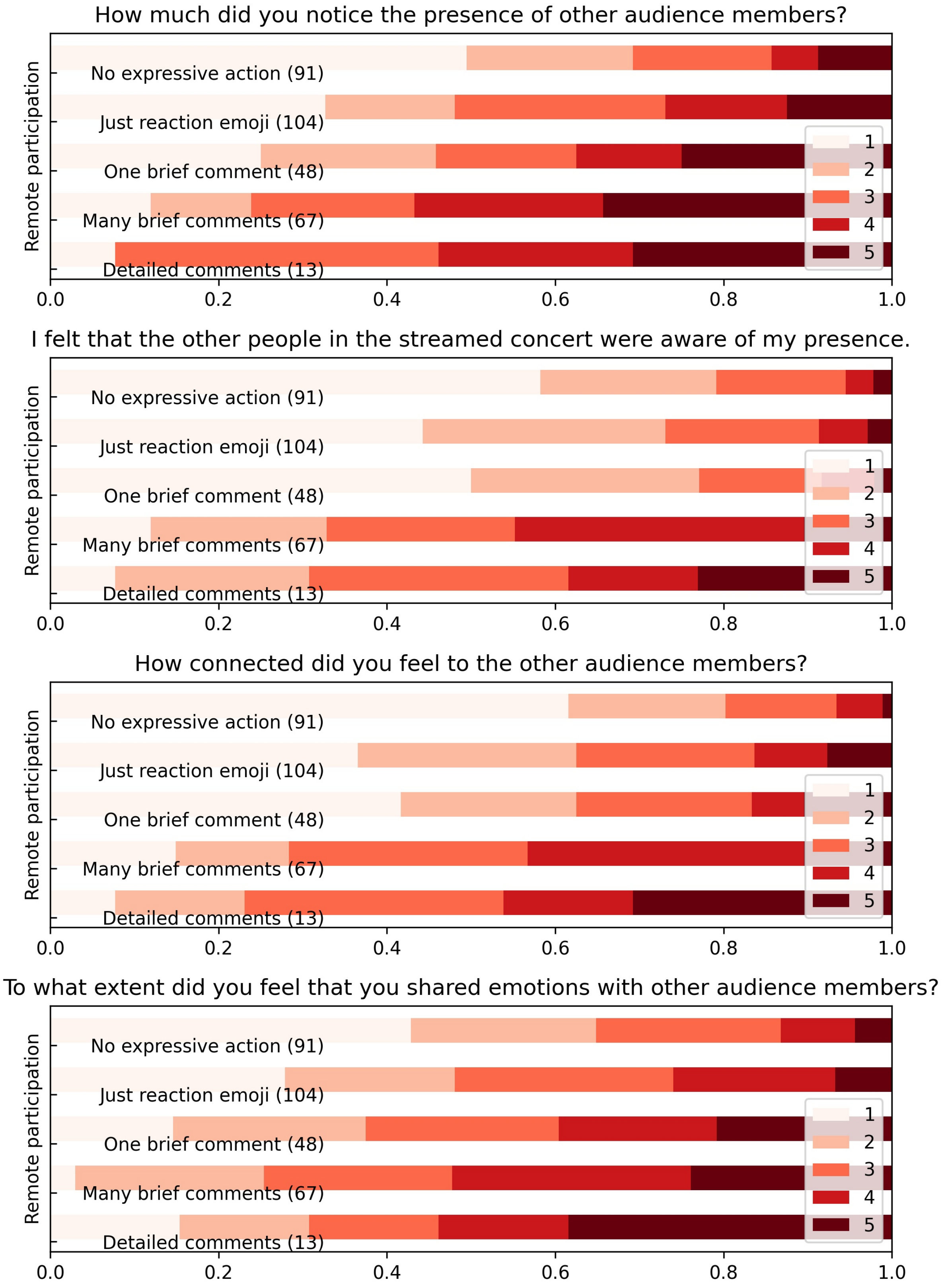


Figure S4. How participants related to others remote audience members, by degree of remote participation.
